# Could the war disruption in Ukraine move micromobility forward? Stakeholders’ perspective

**DOI:** 10.1186/s12544-025-00742-9

**Published:** 2025-09-30

**Authors:** Mariia Olkhova, Asya Natapov, Olha Plyhun, Taimaz Larimian, Dmytro Roslavtsev

**Affiliations:** 1 School of Architecture, Building and Civil Engineering, Loughborough University, Epinal Way, Loughborough, LE113TU UK; 2 Transport Systems and Logistics Department, O. M. Beketov National University of Urban Economy in Kharkiv, 17 Chornoglazivska St, Kharkiv, 61002 Ukraine; 3 Faculty of Environment, Science and Economy, University of Exeter, Prince of Wales Road, Exeter, EX4 4SB UK

**Keywords:** Micromobility, Cycling, Stakeholders interview, Travel behaviour, War impact, Sustainable transport

## Abstract

This study explores the potential for micromobility expansion in Ukraine, viewing the war’s disruption as an opportunity to rebuild urban space and transportation systems and to shift focus from post-Soviet planning to more sustainable urban mobility, prioritising cycling and other active modes. It aims to identify the impact indicators of micromobility usage during the ongoing war and to outline the challenges and prospects for micromobility. Employing a qualitative research approach, semi-structured interviews were conducted with micromobility stakeholders across 16 administrative divisions in Ukraine. The findings shed light on the factors that either hinder or support micromobility users, the importance of collaboration among city stakeholders, and the micromobility role during and after the war. The study revealed regional differences in micromobility stakeholders’ cooperation and infrastructure development, positively correlating collaboration strength and micromobility adoption. These insights can inform strategic interventions to promote micromobility in Ukrainian cities, ensuring affordable and accessible mobility solutions while reducing infrastructure costs during wartime and post-war recovery. The interviewees suggest that despite current challenges, there is optimism for increased micromobility adoption in Ukraine, with estimates of 10–30% long-term adoption, potentially rising to 50% by 2050.

## Introduction: prospects for micromobility

The Russian invasion in 2022 exacerbated these challenges, causing extensive destruction of infrastructure and electricity shortages. Transport is the second most damaged sector after housing [75]. Cities in eastern, northern and southern Ukraine, where destruction is severe, have had to reorganise mobility: free public transport services, shelters at bus stops, and bicycle provisions. Meanwhile, western and central cities, less affected by destruction, have experienced significant population growth due to displacement (1.4 m in 2022, 5.3 m in 2023 [72]), overloading urban infrastructure, including public transport. It becomes evident that transport systems will need postwar rebuilding to repair damage and absorb changes caused by the war. Moreover, this presents a unique opportunity to rebuild the urban spaces and transport systems in Ukrainian cities, transitioning from post-Soviet approaches to sustainable development, prioritising walking, cycling, and other micromobility modes, aligning with the Sustainable and Smart Mobility Strategy in the European Union [16] and the Decarbonising Transport Strategy in the United Kingdom [30]. The basis for this lies in political prospects for Ukraine: granted candidate status in 2022 [21], Ukraine is required to harmonise its legislation with the European Union policies. The 100-year UK-Ukraine Partnership, signed in 2025, also includes commitments to transforming the transport sector [29].

Micromobility has been widely studied across various countries, focusing on sustainability [5, 10, 26, 42, 77], infrastructure development [42], travel behaviour [55, 56], shared mobility [13], user satisfaction [37], and transit integration [8, 73], safety [76]. However, research on micromobility in emergencies or conflict situations is scarce [24]. Responses to crises vary – while covid pandemic led to fewer trips in Zurich [41], usage increased in Chicago [1]. Although military conflicts persist worldwide, there is little research on micromobility usage during or after wars. Research on micromobility in Ukraine remains limited to policy briefs on e-scooters [68] and bicycles [64], NGO reports [11], and journalistic publications [67], none of which offer a systematic analysis of infrastructure, challenges, or war impact. No peer-reviewed studies have been identified in Scopus or related academic databases. Given the scientific achievements and best practices of the United Kingdom and the European Union analysed in [58] – there is significant potential for micromobility development in Ukraine. However, planning must account for the distinct war impact.

In this study, micromobility refers to bicycles, e-bikes, e-scooters and other types of light electric personal transport, whether privately owned or rented, for personal or commercial use.

This research aims to explore the key factors of micromobility usage during the ongoing war and to outline the challenges and prospects for its development in Ukraine. The study offers the first comprehensive analysis of micromobility in Ukraine, focusing on the war impact. These insights can inform strategic interventions to promote micromobility, ensuring affordable and accessible mobility while minimising infrastructure costs during and after the war. Considering the influence of European Union and United Kingdom policies on Ukraine’s transport development, the next section examines their historical narrative of micromobility in more detail.

This paper is structured into six sections. Section 2 outlines the historical development. Section 3 describes the methodology. Section 4 presents the findings, followed by discussion and directions for future research in Sect. 5. Conclusions are provided in Sect. 6.

## Background: practices across the European union, the United Kingdom and Ukraine

Cycling spans two centuries, originating with the bicycle’s invention in 1885. The 1890 s marked the “bike boom”, making cycling widely accessible through clubs and competitions in Britain [7] and other Western countries [44]. In Ukrainian cities, the cycling boom peaked between 1894 and 1902 [44]. However, with the rise of the motorcar in the early 20th century, cycling declined and became associated primarily with leisure [7], losing appeal among elites in Ukraine [44].

Despite this decline, bicycles remained a critical transport mode between World War I and the 1950 s, especially during fuel shortages and in areas with damaged infrastructure, serving both military and civilian mobility needs [51, 62]. In the Union of Soviet Socialist Republics, bicycles were not personal property but symbolically state-owned until the 1950s. Despite increased production, cycling was a low priority in Soviet transport planning, as focus shifted to motorisation and space exploration [45].

At Ukraine’s independence (1991), there was economic decline, bicycle production decreased, and the country inherited urban spaces that were not at all equipped for cyclists [66]. In contrast, from the 1990 s, Europe began systematically integrating cycling into urban transport policies, prioritising congestion reduction and cyclist safety [14, 74]. A significant milestone was the launch of the EuroVelo network in 1994, designed to integrate national and regional cycling routes [22]. Today, EuroVelo4 passes through Ukraine, from the Polish-Ukrainian border to Kyiv, but remains under development [23]. Similarly, the United Kingdom launched the National Cycling Strategy (1996) to double cycling rates by 2002 and quadruple them by 2012 [31]. Although targets were unmet, significant progress occurred in developing cycling infrastructure, including bike paths, rental schemes, and network planning [36]. These initiatives demonstrate how induced traffic and environmental problems have catalysed shifts toward sustainable urban transport, offering relevant lessons for Ukraine’s contemporary policymaking [51, 74].

Cycling infrastructure in Ukraine only began to attract municipal attention in the 2010 s [47]. Vinnytsia pioneered this effort in 2011, followed by Lviv, Ivano-Frankivsk, and Poltava, each implementing cycling development programmes. In 2018, Ukraine approved the National Transport Strategy, which set goals for developing cycling infrastructure through 2030 [70].

Meanwhile, the European Union consistently promoted cycling policies. The White Paper on Transport (2011) recognised cycling as essential for sustainable urban mobility, and the Low Carbon Mobility Strategy (2016) highlighted walking and cycling as key elements of local urban transport plans [17, 20]. The European Cycling Strategy (2017) called for improved infrastructure and a unified European Union cycling policy [15]. In 2020, the Stockholm Declaration further advanced active mobility [28]. However, national cycling plans were not widely prioritised until the Pan-European Master Plan in 2021 [18]. In 2023, the European Parliament called for an EU-wide cycling strategy [2], leading to the adoption of the European Declaration on Cycling (2024) – the first official cycling policy, containing 8 principles and 36 commitments to promote cycling development [19]. In the United Kingdom, the Transport Decarbonisation Plan (2021) ambitiously aims to shift half of urban journeys to walking or cycling by 2030 [30]. The consistent trajectory in the European Union and the United Kingdom highlights crucial policy coherence and sustained investment, which Ukraine can emulate in its efforts to rebuild urban transport systems following the current war.

**Fig. 1:**
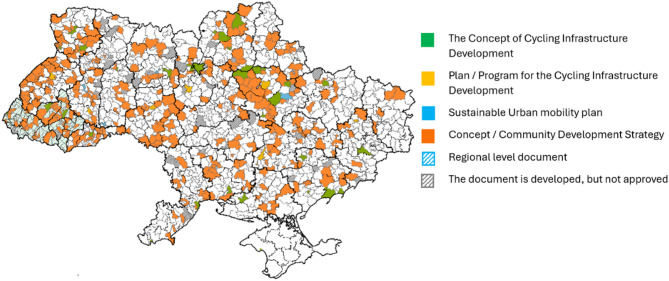
Map of cycling infrastructure development plans in Ukrainian administrative divisions [47]

Importantly, in the European Union and the United Kingdom, sustainable transport policies have been coordinated nationally, unlike Ukraine, where cycling popularity relies largely on NGO advocacy such as U-cycle, Velovector, Velokryvbus, and LAV. Despite these NGO efforts, only 27% of Ukraine’s administrative divisions had approved strategic cycling development plans by March 2024, and there were no suburban, intercity, or inter-rural cycling routes [4, 47] (Fig. 1).

Beyond bicycles, micromobility options such as electric kick-scooters have become more popular. E-scooter trials started in Paris in 2018 [32], raising safety concerns, and there remains no common European Union regulation policy for micromobility. Meanwhile, the United Kingdom began shared e-scooter trials in 2020, though private usage remains illegal [52]. In Ukraine, the first bicycle rental NextBike started in Lviv in 2015, followed by Bolt e-scooter rentals in 2019. By 2021–2022, 26 e-scooter providers operated in Ukraine. In 2023, a new law classified e-scooters, e-bicycles, and other light electric vehicles into two categories: light personal e-vehicles (up to 25 km/h) and low-speed light e-vehicles (10–50 km/h) [71]. However, specific traffic regulations for e-scooters remain undefined. Following this tendency, a significant chance exists for a new “bike/e-scooter boom” in cities, driven by ongoing policies and the growing emphasis on sustainability, along with economic crises, wars, and other potential challenges.

The full-scale Russian invasion of Ukraine has significantly altered travel behaviour, with widespread destruction of transport infrastructure reshaping passenger, freight, and pedestrian flows and micromobility usage [50]. Consequently, four e-scooter operators exited Ukraine in 2023, followed by five more in 2024. However, cycling has emerged as a resilient and increasingly popular transport mode under war conditions [65]. The ongoing war in Ukraine offers a vital opportunity to reshape transport policies towards micromobility as part of sustainable urban recovery. These lessons highlight the effectiveness of policy coherence, stakeholder collaboration, and ongoing infrastructure investment in promoting micromobility, which are crucial considerations for Ukraine’s future planning. Therefore, analysing these comparative historical trajectories not only contextualises Ukraine’s current challenges and prospects but also underscores the strategic potential for transformative urban recovery aligned with broader European sustainability commitments.

This study explores the challenges of expanding micromobility usage in war-damaged Ukrainian cities and its impact on travel behaviour. The research questions include:

- What are the key factors influencing micromobility usage in Ukraine?
- How does stakeholder cooperation relate to the development and usage of micromobility infrastructure in Ukrainian cities?
- How has the war impacted travel behaviour and micromobility infrastructure in Ukraine?

## Methodology: gathering stakeholders views in Ukraine

**Fig. 2:**
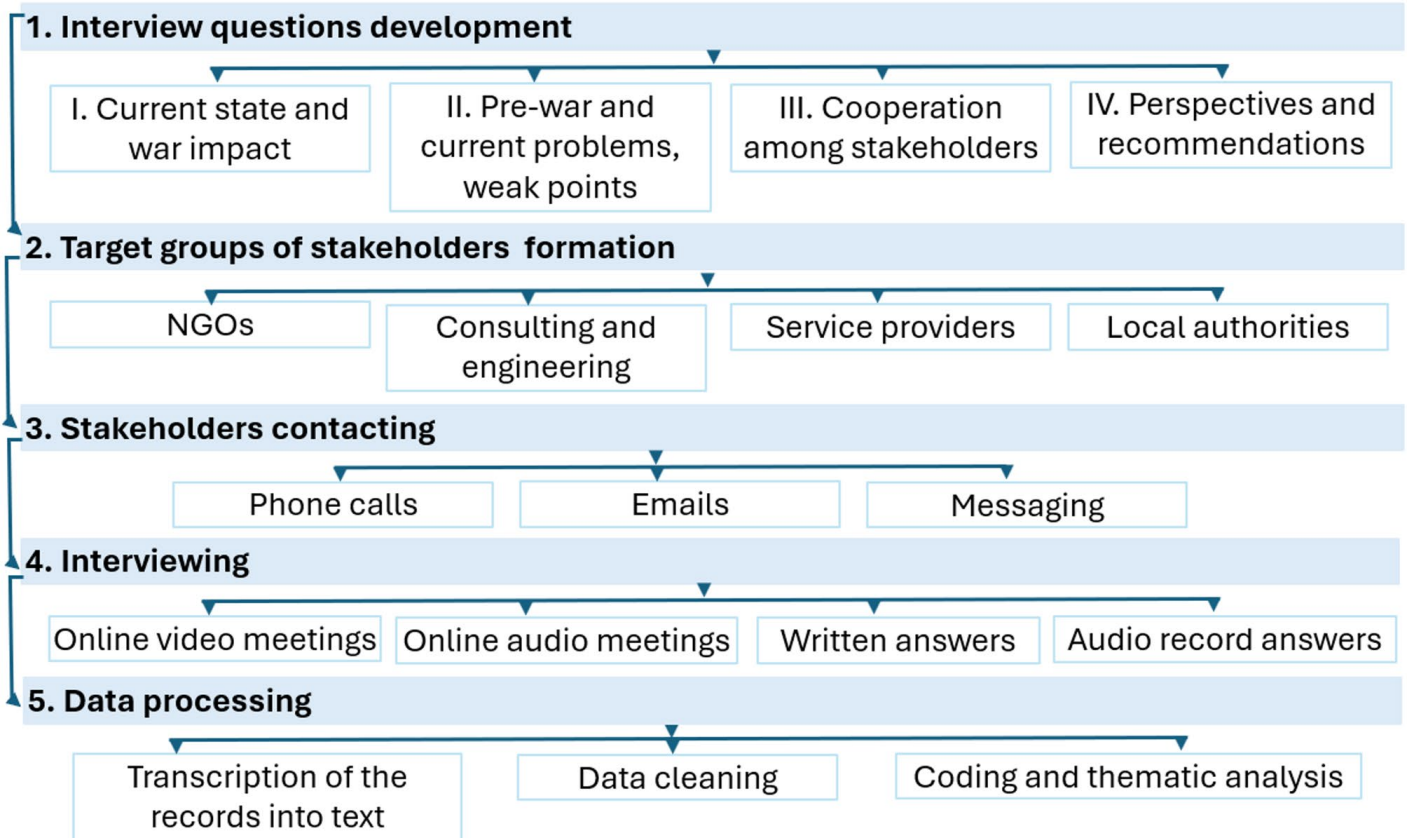
Methodology of stakeholders interviewing

To outline the general state of micromobility, open-source data was analysed, including the National Transport Strategy of Ukraine 2030, analytics from the Ministry for Development of Communities and Territories of Ukraine, Derzhstat national statistics, the websites of the 24 administrative divisions in Ukraine, service provider websites, NGOs contributions, professional blogs and social media and network channels, and relevant legislation on cycling and personal light transport. Afterwards, semi-structured interviews were conducted with stakeholders to identify their perceptions, war impact on travel behaviour, challenges, and prospects for micromobility in Ukraine. The interviewees were contacted through phone, email, and messaging (Fig. 2).

### The interview questions

The interview questions were organised into four blocks: current state and war impact; pre-war and current problems, weaknesses; cooperation among stakeholders; perspectives and recommendations. Each block contained of 3–6 questions, tailored slightly for different stakeholder groups based on their expertise. Responses were analysed according to four thematic areas. The study protocol was reviewed and approved by the Transport Systems and Logistics Department at O. M. Beketov National University of Urban Economy in Kharkiv, ensuring compliance with ethical standards for research involving human participants. Informed consent was obtained from all participants, who received a detailed information sheet outlining the study’s purpose and procedures.

### A target group of stakeholders

The stakeholder groups were categorised based on their role in micromobility development: (1) local authorities, (2) non-governmental organisations (NGOs), (3) consulting and engineering companies involved in transport infrastructure design, and (4) micromobility service providers. Initially, food delivery companies were considered but later excluded, as they do not mandate micromobility use; couriers independently choose their mode of transport. Local authorities include departments responsible for transport, infrastructure, architecture and urban planning, and their structures vary by city, posing challenges. NGOs represent micromobility users, advocating for sustainable mobility and road safety, offering in-depth insights into micromobility users through surveys and independent analytics. A database was compiled, listing stakeholders across different regions, including their websites, social media, emails, phone contacts, key personnel, and geographical distribution. Based on publicly available information, 114 organisations were identified as potential interviewees, distributed as follows: 13 representatives of local authorities (Lviv, Kharkiv, Kyiv, Rivne, Chernihiv, Ivano-Frankivsk, Poltava, Vinnytsia, Mykolaiv, Khmelnytskyi, Chernivtsi, Myrhorod, Dnipro); 5 consulting and engineering companies (ProMobility, Kyiv; Egis, Lviv; RS Engineering Kharkiv; Urban Progress, Ivano-Frankivsk; Viasystempro, Kharkiv); 6 micromobility rental service representatives (Bolt, NextBike, Jet Sharing, Strum, Vevi, Prokataisia); 10 NGOs (U-cycle, Spilno Hub, Veloboiarka, Velokruvbas, Velovector, LAV, Prostir Zlagoda, Ecomisto, Urban Crew, Urban Dnipro).

### Communication with stakeholders and interviewing

We approach 114 organisations via email, social media (Facebook, Instagram), the university’s website, and direct communication through calls and messaging apps (Telegram, Viber, WhatsApp). Five NGOs were found to be inactive, 68 stakeholders did not respond, and 8 formally declined participation. The response rate was 30%, with 70% declining participation (7% providing rejections). The ongoing war likely contributed to this high rejection rate, as many organisations, especially in eastern, northern and southern Ukraine, have suspended operations or shifted to volunteering. Interviews covered 16 of the 24 administrative divisions (67%).

The interviews lasted from August–October 2023, with an average duration of one hour. The primary way to conduct the interview was a video/audio meeting. Most were conducted via video or audio calls, though respondents could also submit written answers or audio recordings. Data was collected from 34 respondents, including one written response and one audio recording.

### Processing of the collected data

The interviews were conducted in Ukrainian and transcribed using Notta.ai as Zoom and Teams do not support Ukrainian. Following transcription, the data underwent a cleaning process that involved verifying the automatically generated transcripts and expert verification to identify and correct errors. Common transcription inaccuracies, such as homophones, incorrect punctuation, and grammatical inconsistencies, were rectified. Technical terms related to micromobility were cross-referenced with industry standards for accuracy, particularly in alignment with the Network on European Communications and Transport Activities Research. A uniform formatting style was also applied across all transcripts, standardising font type, size, and line spacing. Non-relevant content, such as filler words, off-topic discussions, and background noise, was removed to streamline the transcripts and focus on the study’s core subject matter.

Data analysis followed a systematic coding process using NVivo14. Initially, we created 138 codes, which we reviewed to identify patterns, consolidate related codes, and group them into broader themes. A thematic content analysis was applied using an inductive approach to identify commonalities, differences, and interrelationships among the codes [12, 63]. Once the preliminary themes were established, a review was undertaken to ensure logical coherence with the research questions and provide a robust analytical framework in line with the methodology outlined in [6, 48]. Seven themes were identified: micromobility in Ukraine, challenges, stakeholder collaboration, shared micromobility, sustainability perspectives, war impact, and postwar development.

## Findings

### Overview of micromobility in Ukraine

The first section of the interview “General state: pre-war and current state”, included the following questions: What micromobility modes were used in cities before the war, and which are currently in use? Which micromobility modes do you believe hold the most promise? Rank them based on anticipated demand. Describe the trends in demand for micromobility in the short and long term. What percentage of trips do you think will be made using micromobility in the future?

The most frequently mentioned micromobility modes were bicycles, e-scooters and e-bikes, while hoverboards and monowheels were also noted. Most respondents reported a marked increase in e-scooter demand prior to the invasion, which generally continues, except in frontline areas where rental services have been suspended or reduced.

**Fig. 3:**
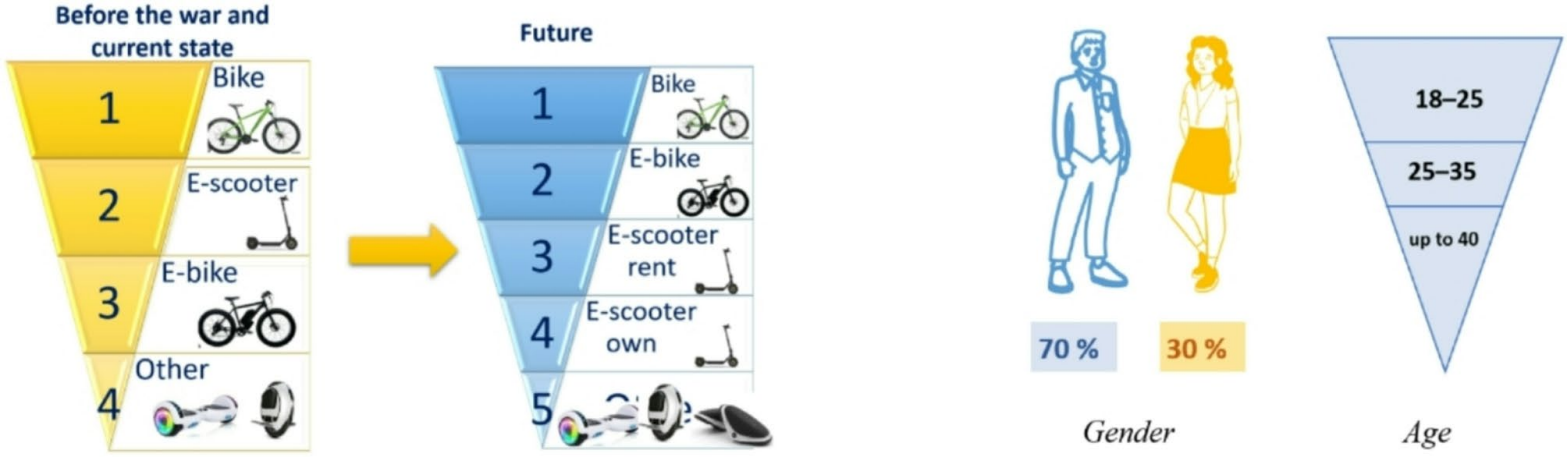
Vehicles and users of micromobility: stakeholders’ perspective

Looking ahead, the majority of respondents indicated a slight shift in focus towards e-bikes. Additionally, bike rentals, personal and rental e-scooters were identified as promising options (Fig. 3).

**Fig. 4:**
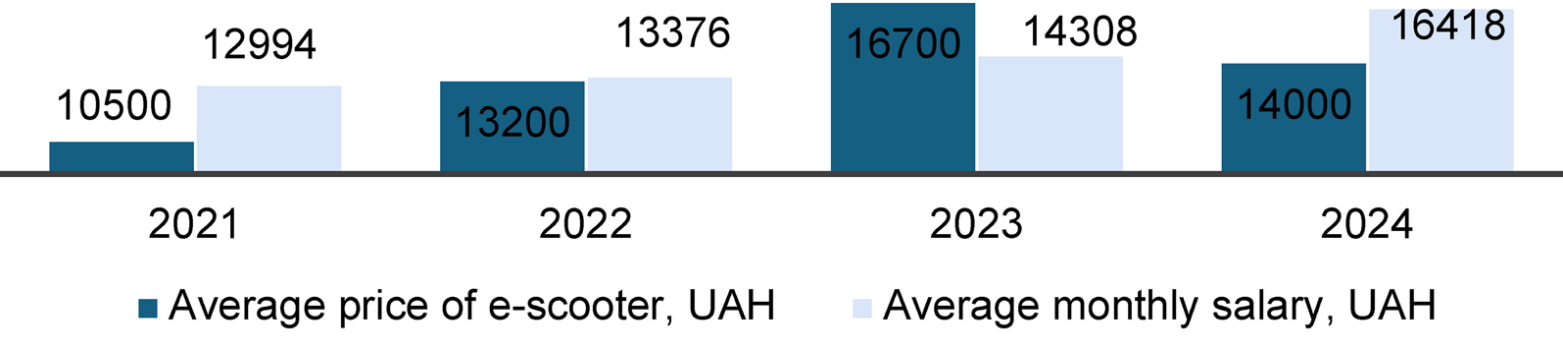
E-scooter price and official monthly salary in Ukraine

Figure 4 – Vehicles and users of micromobility: stakeholders’ perspectiveFour representatives of e-scooter and bike providers, based on their annual business reports, estimated that 70% of micromobility users are male, while 30% are female. Users are typically aged between 18 and 40, with the majority falling within 18–25.

Regarding the anticipated share of future trips, not all respondents could provide precise estimates due to the complexities of long-term forecasting. However, eight responses from NGOs suggested that, optimistically, 10–30% of the trips could be made by using micromobility in the long term. One respondent projected that, during the warmer months, this share could increase to 50% by 2050. Only two representatives from service providers assumed that 30–40% of trips could be made using micromobility modes in the future, while one engineering company predicted just 5% in the long-term.

All service providers reported that e-scooters and bicycles are primarily used for commuting, household-related travel, and leisure. Micromobility also functions as a last-mile solution, improving connectivity between public transport and final destinations.

### Factors influencing micromobility usage in Ukraine

The second set of interview questions focused on challenges, weaknesses and obstacles to micromobility development before and during the war. This section addressed the initial research question: *What are the key factors influencing micromobility usage in Ukraine?*

**Fig. 5:**
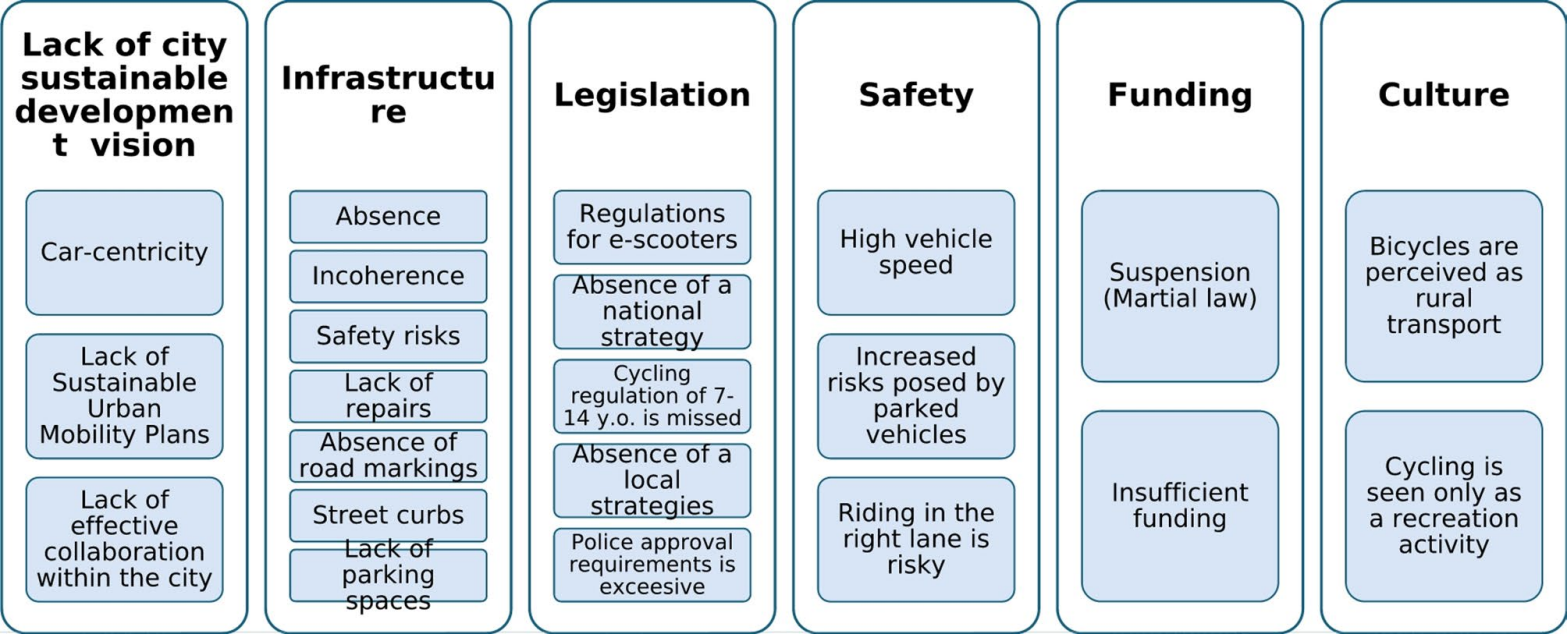
Micromobility challenges in Ukraine

All participants unanimously identified the lack of infrastructure as the most significant constraint (Table 1; Fig. 5).

**Table 1:** Constraints and facilitators for the micromobility users in Ukraine

Constraints	Facilitators
Lack of infrastructure	Availability and quality of infrastructure
Infrastructure quality: incoherence	Creating safe driving conditions
Danger on the roads	Availability of rental services
Lack of bicycle storage space	Public transport: high fare, lack of availability
Cost of micromobility vehicle	High fuel price
Perception of the bicycle as rural transport	Warmer winter weather conditions

Safety concerns were identified as the second major limiting factor indicated by 30 interviewees: 1502 accident cases with cyclists in 2024 (3.8% increase), 175 fatalities, and 1395 injuries [54]. Additionally, the shortage of parking spaces as noted as another critical limitation by all respondents, except the service providers.

Another constraint mentioned by all NGOs and the majority of local authorities was the high price of bicycles and e-scooters. To explore this issue, Fig. 4 compares the average salary and price of e-scooters in Ukrainian currency. The figure clearly demonstrates that e-scooters are expensive, approximately equal to the average monthly salary in Ukraine. Even a used e-scooter costing around half of a monthly salary.

Among the key facilitators for micromobility usage were infrastructure availability, quality, and safety. The presence of rental services also significantly enhances micromobility acceptance. High public transport fares and rising fuel prices push users towards seeking alternative transport modes. Additionally, the trend of warmer winters, mentioned by NGOs, has extended the period during which micromobility can be used in Ukraine.

Representatives from rental services noted that demand for e-scooters is influenced by various factors, including seasonality, weather, city development, traffic conditions, availability of docking stations, fares, public transport costs, road quality, the availability of cycling paths, and access to public transport. They also mentioned that theft rates are not higher than the average in European countries. Two representatives of local authorities and one service provider indicated that the demand for rental bicycles has significantly declined since the introduction of e-scooters. Respondents attributed this to e-scooters being free from docking stations, easier to operate, and more appealing. These findings align with the central NGO U-Cycle analytics [65], which shows an increasing number of shared users from 2020 to 2022: e-bikes rose by 0.16%, while e-scooters increased by 20%. To summarise respondents’ insights, six directions of the challenges were highlighted (Fig. 5).

In addition to infrastructure, regulatory policy challenges remain significant, especially the need to formally recognise micromobility users as a distinct category of road users in traffic regulations. NGO interviewees highlighted key safety concerns for cyclists, including high-speed traffic (up to 70 km/h without fine) and visibility issues caused by parked vehicles, which restrict manoeuvrability for users.

All local authorities noted that limited funding is further constrained for development, as capital expenditures were suspended under Martial law. Additionally, bicycles are still largely perceived as rural or recreational transport, due to inadequate infrastructure and the low visibility of cycling activity in cities.

### Cooperation among stakeholders

**Fig. 6:**
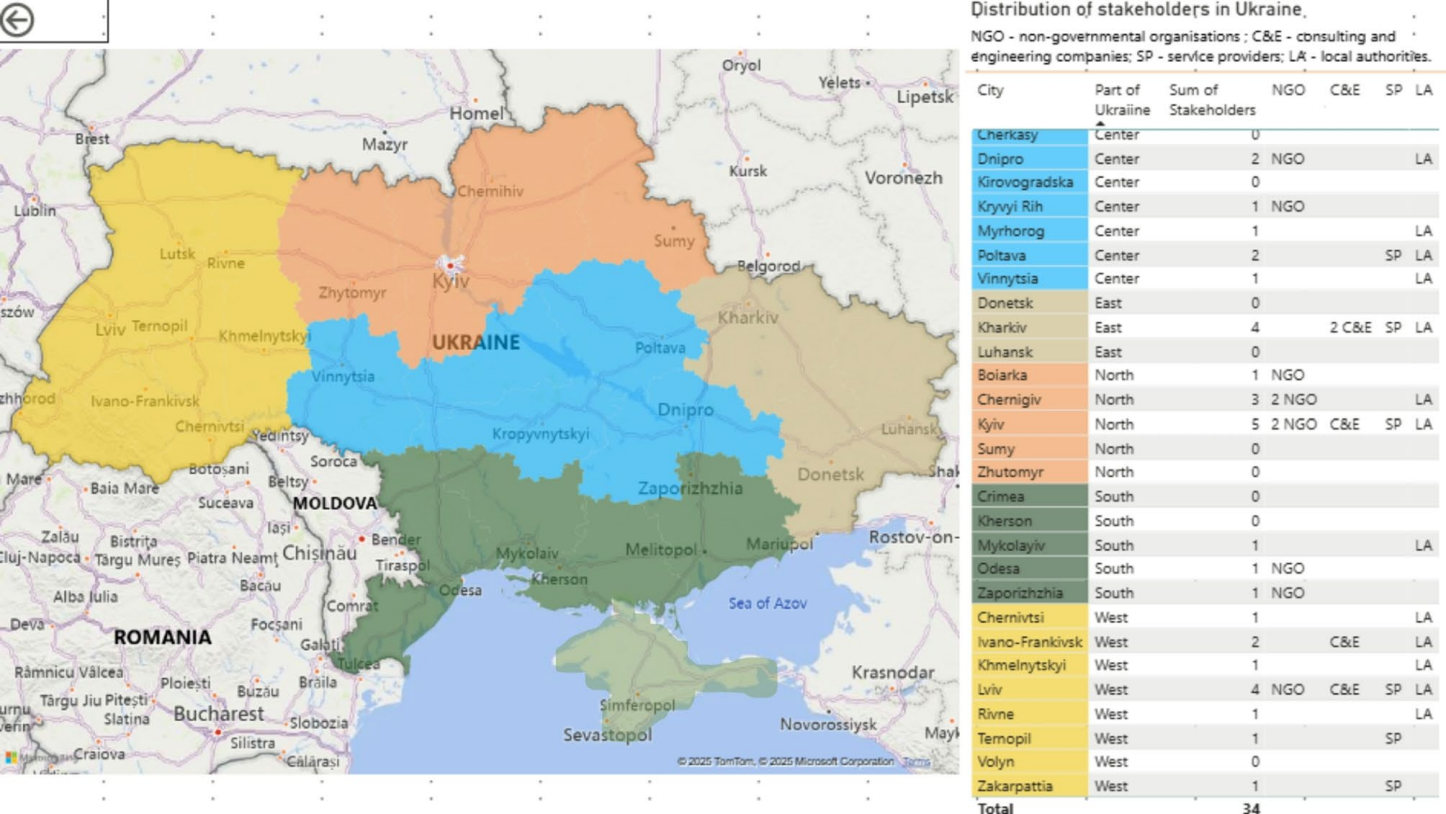
Map of Ukraine: stakeholders’ involvement

Considering the various levels of existing cycling infrastructure in Ukrainian cities from analytics in Sect. 2, it was assumed that the relationship between stakeholders’ cooperation and micromobility infrastructure should be explored (Fig. 6). This addressed the second research question: *How does stakeholder cooperation relate to the development and usage of micromobility infrastructure in Ukrainian cities?*

Each respondent was asked about stakeholder cooperation. Local authorities were questioned: How should cooperation with NGOs and local authorities be organised? Can they assist in the development of micromobility? Service providers, as well as consulting and engineering companies, were asked the same questions but with a focus on cooperation between them and local authorities. A second set of questions examined stakeholder influence: What influence do NGOs, local authorities and businesses have on micromobility in cities? Do NGOs and businesses contribute to the development of micromobility, and if so, how? What are the problems in cooperation?

The findings highlight notable differences in transport system planning approaches regarding micromobility. The following tendencies have emerged:

- Established cooperation between local authorities and NGOs.
- Initiation of collaboration with service providers, formalised through memorandums.
- Effort to involve stakeholders in the discussions.
- Reliance on personal connections, with cooperation needing to be rebuilt when contacts are lost.
- Limited engagement NGOs with local authorities, where decision-making is confined to a small group of officials.

Stakeholder collaboration varies significantly across Ukraine. As stated by all local authorities, NGOs play a crucial role in advancing micromobility by developing strategies and plans, sharing best practices, and advocating for users. However, this cooperation tends to be bureaucratic isolation in the eastern, northern, and southern parts of the country. In these areas, promoting cycling infrastructure is often met with scepticism. This is demonstrated by a statement from a representative of an NGO in southern Ukraine, who mentioned their local authorities’ stance: “If 50 cyclists pass through, then we will consider constructing a cycling path”. This mindset reinforces a cycle of low micromobility adoption, as poor infrastructure discourages usage, making demand less visible [25]. It also reflects outdated Soviet-era transport planning, characterised by centralised decision-making, high curbs, and a lack of stakeholder engagement and sustainable vision [3, 61].

Collaboration between rental services and local authorities is generally formalised through memorandums regulating permitted speeds (20 km/h and 15 km/h), parking regulations, and other operational aspects. Respondents noted that cooperation is more transparent and effective in urban areas with better infrastructure, mainly in western and central Ukraine, involving diverse stakeholders and strong feedback mechanisms in micromobility planning. A geographical analysis of responses reveals a clear pattern: the strength of stakeholder cooperation – particularly with NGOs – correlates positively with infrastructure development and the number of users. This trend follows a westward trajectory across the country, transitioning from regions with limited cooperation to those with more robust collaborative frameworks. For infrastructure evaluation, we combined responses from different stakeholder groups and examined official data [59]. The absolute length of cycling infrastructure doesn’t objectively measure micromobility development, as city sizes and road networks vary. By calculating the ratio of cycling length to road length, we identified the top Ukrainian cities in micromobility: Lviv (1.3%), Kyiv (1.1%), Vinnytsia (0.7%), Ivano-Frankivsk (0.4%), Chernivtsi (0.5%), and Chernihiv (0.4%).

### War impact on travel behaviour and transport infrastructure

The Russian invasion of Ukraine has triggered large-scale migration, forcing residents near the borders to flee amidst gunfire and explosions. In frontline regions such as Kharkiv, Sumy, Chernihiv, Kherson, and Kyiv, people queued for essential supplies at grocery stores and fuel stations. During the first month, mass displacement occurred, with many relying on private vehicles or organised evacuation via trains and buses. The war has profoundly disrupted daily life, including mobility patterns. This led to the third research question: *How has the war impacted travel behaviour and micromobility infrastructure in Ukraine?*

#### Travel behaviour changes

An analysis of the respondents’ answers revealed significant changes in the transport system due to the war, with regional variations in impact. Respondents’ answers were categorised geographically, dividing the country into regions: east, west, south, north and centre (Table 2).

The respondents from frontline areas observed a growing reliance on bicycles in small and medium-sized cities where public transport services have been reduced or are unavailable. In these areas, bicycles enable residents to save both time and money. “In some places, it was the only one mobility mode for the population”, and “people picked up all the old bikes”. According to service providers, in larger and medium-sized cities, e-scooters are predominantly used by internally displaced youth, primarily due to limited access to private vehicles.

**Table 2:** War impact on the transport system and travel behaviour in Ukraine

Change	Part of country
The intensity of traffic has decreased	East, south, north
Lack of public transport	East, south, north
Reduction of public transport service	East, south, north
The number of cyclists has increased	East, south, north, centre
The bicycle became the only possible means of mobility	East, north
Increase taxi services during an active invasion by 20 times	East
The bicycle sharing service has closed	South, north
The number of cars increased, and traffic jams appeared	West, centre
No significant changes in micromobility usage were observed	West
Population growth	West
Block posts as a reduction in traffic intensity	Overall Ukraine
Moving the rental service from the frontline cities	Overall Ukraine
Internally displaced youth intensively use micromobility	Overall Ukraine
Lack of drivers for public transport	Overall Ukraine
The general mobility of the population has decreased	Overall Ukraine
Lack of drivers because of the decrease in the male population	Overall Ukraine

Additionally, the surge in micromobility usage has been driven by the fuel crisis, caused by the destruction of fuel facilities. However, during electricity outages, respondents highlighted the distinct advantages of mechanical bicycles, emphasising their reliability as a transport mode in unpredictable situations. This trend is particularly evident in liberated regions, where Ukrainian troops have reasserted control. The population in these areas predominantly comprises older individuals or those with lower socioeconomic status. It is also noteworthy that, during the occupation, Russian troops confiscated bicycles from the local population, further exacerbating mobility challenges in these regions. A similar behaviour was exhibited by Nazi rule during World War II [44].

#### Impact on infrastructure destruction

The impact on the transport, road, and energy sectors across different regions was examined to comprehensively understand how the ongoing war has affected these critical infrastructures, highlighting regional disparities and specific challenges faced in each area (Table 3, see Fig. 6).

**Table 3:** War impact on transport infrastructure in Ukraine

Infrastructure impact	Reason	Region
A complete stop of public transport	Period of active invasion, danger	East, centre, south, north
Lack of bus, trolleybus transportation	Complete destruction of the fleet	South, north
Partial operation of the metro	The metro is used as a shelter	East, north
Increasing the traffic interval of public transport (buses, trolleybuses, trams)	Partial or complete destruction of public transport and energy system destruction	East, south, centre, north
Road destruction (car, bicycle paths)	Missile and artillery fire	East, south, north
Public transport suspension during air alarms	Danger of shelling	East, south, north
Capital repairs prohibition, only emergency repairs	Martial law and finance limitation	Ukraine
Freezing infrastructure projects	Martial law and finance limitation	Ukraine

#### Micromobility during the war

Representatives of NGOs and local authorities highlighted the challenges in promoting cycling education and awareness in cities due to restrictions imposed by Martial law and safety concerns. Representatives of local authorities noted the problem of a shortage of engineering personnel and male drivers. Additionally, most cities have curfews from 10pm–5am, restricting movement. Thus, rental operators noted a shift towards e-scooters as an alternative to taxis in the evenings. The issues for business include the inability to conduct nighttime maintenance (charging, repairs, redistribution), electricity outages, and communication disruptions with e-scooters due to air raids and Russian electronic warfare, particularly in the eastern regions.

### Perspectives of micromobility development

**Fig. 7:**
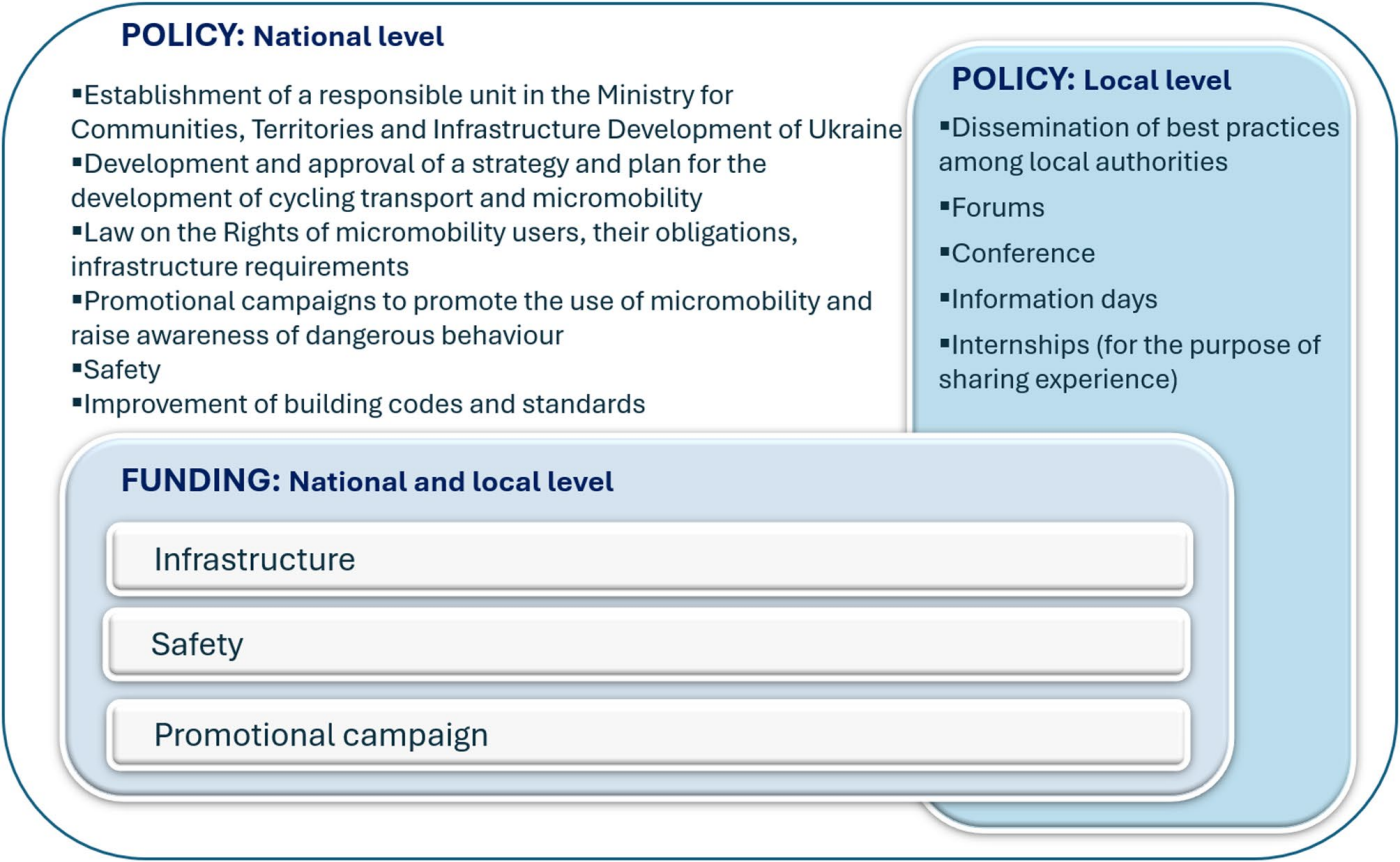
Changes needed for micromobility advancement: stakeholders’ perspective

Despite the devastating challenges and adverse consequences that the war continues to inflict, all respondents have observed a potential positive impact on the future expansion of micromobility in Ukraine. They attribute this optimism to an increased public awareness of micromobility importance, a rising demand for bicycles, and growing citizen influence on municipal leadership. One respondent from engineering emphasised: “People want change, and leaders understand this and cooperate”. To drive this transformation, national-level reforms are essential, starting with the establishment of a comprehensive state policy to promote cycling across Ukraine. As one participant from NGO noted, “Our only way forward is sustainable urban mobility. We should learn from the mistakes European cities made 30–40 years ago to avoid repeating them”. A synthesis of responses regarding the essential changes needed for micromobility advancement at the national level is shown in Fig. 7.

Respondents emphasised the importance of collaboration among city authorities and the business sector. This involves enhancing public engagement to ensure joint decision-making, adhering to subsidiarity, and decentralising the budget. A practical model for cooperation involves integrating public transport with rental services, enabling users to purchase a single transport ticket and receive incentives, such as bonuses, for using bicycles or scooters. This approach promotes multimodal transport and encourages wider adoption of micromobility.

## Discussion

Despite the highly complex challenges facing Ukrainian cities, it is vital to develop a holistic, long-term vision for their reconstruction, adopting new approaches to create attractive urban spaces [57]. Postwar rebuilding presents a unique opportunity to establish inclusive and efficient transport systems that serve all users, including pedestrians, cyclists, passengers, cars, motorists and people with disabilities. Achieving this requires a shift in urban planning to prioritise users’ needs. Micromobility development presents an opportunity to move forward with a sustainable city transport system [10, 35].

Our interviews have shown that war’s devastation provides a chance to rebuild Ukrainian cities, focusing on sustainability and resilience – thereby creating new mobility for society – incorporating micromobility to enhance urban transport, improving accessibility and affordability, and following safety recommendations in [34]. Furthermore, this will support the integration of Ukrainian cities into the Trans-European Transport Network (EuroVelo), enhancing intermodality, for instance, by linking micromobility with railways.

The war impact on urban mobility is a complex multi-criteria and multi-stakeholder issue, varying significantly across regions. For instance, our findings reveal differing opinions: while eight respondents reported no change in their cycling and e-scooter usage, 26 noted an increase, highlighting the need for further investigation. Despite this discrepancy, common themes emerged: all interviewees observed rising micromobility use during the last years, raised concerns about inadequate infrastructure, and emphasised the need for collaboration between stakeholders and postwar rebuilding. The interviewees also demonstrated varying expertise: local authorities focused on both cycling and e-scooters, NGOs deepened into cycling issues, and service providers emphasised more e-scooter regulation. This diversity provides valuable insights into Ukraine’s micromobility landscape and identifies areas for future research. Another example is that war impacts also vary regionally. In frontline and de-occupied areas, the destruction of transport infrastructure has increased reliance on bicycles as a primary mode, mainly due to the lack of public transport. Additionally, the fuel crisis, triggered by the destruction of energy facilities, has further driven micromobility adoption as residents seek alternatives to car ownership. Conversely, the war has underscored the advantages of private vehicle ownership, especially for a quick and safe evacuation, that can have a lasting impact on travel behaviour, potentially limiting the growth of shared transport services and micromobility. Thus, any strategy to encourage micromobility adoption must account for these diverse regional challenges.

Despite limited financial resources and time for decision-making, the destruction presents a unique opportunity to adopt a “build back better” approach [40], transitioning from outdated, post-Soviet urban planning towards sustainable development. This requires moving beyond the outdated post-Soviet mindset towards stronger stakeholder cooperation. However, collaboration remains complex. While western cities follow Sustainable Urban Mobility Plan guidelines, many others fail to engage stakeholders effectively, leading to car-centric urban planning that neglects accessibility and affordability. Addressing this gap requires national policies promoting sustainable transport aligning with the European Declaration on Cycling [19], the Transport Decarbonisation Plan in the United Kingdom [30], non-motorised transport policy toolkit [69]. The Ministry for Development of Communities and Territories of Ukraine should lead this initiative, ensuring distribution and monitoring. Additionally, fostering exchanges between western Ukrainian regions and international partners can help policymakers adopt more sustainable transport strategies.

Considering the immense potential for micromobility development in Ukraine and its critical role in improving mobility, it is essential to explore its integration with public transport and walking [9, 39, 49], reflecting the wartime shifts in travel behaviour. Strategic development of integrated micromobility and public transport will support sustainable, affordable and accessible mobility while reducing infrastructure costs in the war/postwar period [27, 38, 46]. This would also strengthen resilience against any future threats and facilitate the return of Ukrainians to reside, work and travel in their country.

## Conclusion

This study offers the first detailed analysis of micromobility in Ukraine, highlighting future opportunities based on pre-war trends and wartime impacts. Although Ukraine is behind the European Union and the United Kingdom, noticeable progress is underway. NGOs lead micromobility development, prioritising bicycles and e-bikes based on their expertise. Conversely, service operators focus on e-scooters, driven by global trends and local business opportunities. Interviewees estimate micromobility could account for 10–30% of trips long-term, possibly reaching 50% by 2050. Most users are male (70%) and aged 18–40. Key barriers include poor infrastructure, safety concerns, high costs, and limited parking. Facilitators include infrastructure quality, rental services, rising fuel prices, and warmer winters. Cooperation between local authorities, NGOs, and businesses varies. In eastern, northern, and southern regions, collaboration is bureaucratic and lacks effective engagement, while stronger cooperation in the west correlates with better infrastructure. The war has reshaped mobility, especially in smaller cities with limited public transport and fuel shortages. Cycling and e-scooter use have increased, with mechanical bicycles proving reliable during power outages. Despite Martial law and curfews, respondents foresee continued micromobility growth driven by awareness, bicycle ownership, and public influence on governance.

This study covers 67% of administrative districts but has limitations, including a 30% response rate, which may affect generalisability. Additionally, the study’s focus on qualitative interviews, though insightful, limits the scope for statistical generalisation, and future research could benefit from a mixed-methods approach that combines qualitative and quantitative data. Another limitation is the rapidly changing environment in Ukraine due to the ongoing war, which may affect the relevance of the findings over time. Future research should explore longitudinal studies to capture evolving trends in micromobility usage during and after the war, as well as the impact of specific infrastructure policies on micromobility adoption. Moreover, comparing the Ukrainian context with other post-conflict regions could provide a broader framework for understanding the role of micromobility in urban recovery and resilience.

Findings offer policymakers insights for developing sustainable urban mobility strategies. Transport planners can use regional disparities and stakeholder cooperation patterns to design effective infrastructure. Local authorities can leverage demographic insights to encourage micromobility. By incorporating these findings, stakeholders can work towards creating more sustainable, accessible, and resilient urban mobility solutions that align with both immediate postwar needs and long-term sustainability goals.

## Data Availability

The data supporting this study’s findings are available on reasonable request.
